# Molecular evidence supports simultaneous association of the achlorophyllous orchid *Chamaegastrodia inverta* with ectomycorrhizal *Ceratobasidiaceae* and *Russulaceae*

**DOI:** 10.1186/s12866-020-01906-4

**Published:** 2020-08-03

**Authors:** Lorenzo Pecoraro, Xiao Wang, Giuseppe Venturella, Wenyuan Gao, Tingchi Wen, Yusufjon Gafforov, Vijai Kumar Gupta

**Affiliations:** 1 School of Pharmaceutical Science and Technology, Health Sciences Platform, Tianjin University, 92 Weijin Road, Nankai District, Tianjin, 300072 China; 2 Department of Agricultural, Food and Forest Sciences, University of Palermo, Palermo, Italy; 3 The Engineering Research Center of Southwest Bio-Pharmaceutical Resources, Ministry of Education, Guizhou University, Guiyang, China; 4 Laboratory of Mycology, Institute of Botany, Academy of Sciences of Uzbekistan, Tashkent, Uzbekistan; 5 AgroBioSciences and Chemical & Biochemical Sciences Department, University Mohammed VI Polytechnic, Hay Moulay Rachid, Ben Guerir, Morocco

**Keywords:** Achlorophyllous orchids, Ceratobasidiaceae, Ectomycorrhizal fungi, Endangered species, Orchid mycorrhiza, Plant-fungus interactions, Mycoheterotrophy, *Russula*

## Abstract

**Background:**

Achlorophyllous orchids are mycoheterotrophic plants, which lack photosynthetic ability and associate with fungi to acquire carbon from different environmental sources. In tropical latitudes, achlorophyllous forest orchids show a preference to establish mycorrhizal relationships with saprotrophic fungi. However, a few of them have been recently found to associate with ectomycorrhizal fungi and there is still much to be learned about the identity of fungi associated with tropical orchids. The present study focused on mycorrhizal diversity in the achlorophyllous orchid *C. inverta*, an endangered species, which is endemic to southern China. The aim of this work was to identify the main mycorrhizal partners of *C. inverta* in different plant life stages, by means of morphological and molecular methods.

**Results:**

Microscopy showed that the roots of analysed *C. inverta* samples were extensively colonized by fungal hyphae forming pelotons in root cortical cells. Fungal ITS regions were amplified by polymerase chain reaction, from DNA extracted from fungal mycelia isolated from orchid root samples, as well as from total root DNA. Molecular sequencing and phylogenetic analyses showed that the investigated orchid primarily associated with ectomycorrhizal fungi belonging to a narrow clade within the family Ceratobasidiaceae, which was previously detected in a few fully mycoheterotrophic orchids and was also found to show ectomycorrhizal capability on trees and shrubs. Russulaceae fungal symbionts, showing high similarity with members of the ectomycorrhizal genus *Russula*, were also identified from the roots of *C. inverta*, at young seedling stage. Ascomycetous fungi including *Chaetomium*, *Diaporthe*, *Leptodontidium*, and *Phomopsis* genera, and zygomycetes in the genus *Mortierella* were obtained from orchid root isolated strains with unclear functional role.

**Conclusions:**

This study represents the first assessment of root fungal diversity in the rare, cryptic and narrowly distributed Chinese orchid *C. inverta*. Our results provide new insights on the spectrum of orchid-fungus symbiosis suggesting an unprecedented mixed association between the studied achlorophyllous forest orchid and ectomycorrhizal fungi belonging to Ceratobasidiaceae and Russulaceae. Ceratobasidioid fungi as dominant associates in the roots of *C. inverta* represent a new record of the rare association between the identified fungal group and fully mycoheterotrophic orchids in nature.

## Background

Mycoheterotrophic plants are associated with fungi that support their heterotrophy with varying extents. Indeed, these peculiar plants acquire carbon through mycorrhizal association with fungal mycelia fetching organic compounds from different environmental sources [1]. Among mycoheterotrophs, achlorophyllous plants, represented by more than 400 vascular species, are fully heterotrophic, since they are not able to photosynthesize and rely solely on carbon from their mycorrhizal fungi [2]. Some plants that contain chlorophyll are partially heterotrophic, because they respire more carbon than they fix and thus obtain part of their organic nutrients heterotrophically via associated fungi and part autotrophically by photosynthesis [3]. In addition, many plants are mycoheterotrophic only during their early stage of development, but become autotrophic when fully developed [1].

The Orchidaceae family is particularly predisposed to mycoheterotrophy, encompassing all known levels of nutritional dependence upon fungi. All orchids are mycoheterotrophic during their establishment phase, since they produce extremely small seeds that lack the nutrient reserves necessary for early growth. For this reason, orchid seed germination and the following achlorophyllous protocorm stage depend on the presence of a fungal partner that provides water, mineral nutrients and organic carbon to the juvenile plant [4]. At the adult stage, most orchid species become fully autotrophic, and although they maintain a relationship with their root associated fungi, this is no longer for carbon nutrition, but just for water and mineral uptake [5]. In contrast, a significant number of forest orchids that develop a photosynthetic apparatus at adulthood remain dependent on both organic and inorganic fungal nutrients under low light availability [6]. This dual nutritional strategy combining mycoheterotrophy and photoassimilation is known as partial mycoheterotrophy or mixotrophy [7]. Recent studies based on stable isotope analysis, including ^2^H, ^13^C, ^15^N and ^18^O, for understanding nutrient exchange between orchids and fungi, have shown that partial mycoheterotrophy plays a far greater role than previously assumed, not only in forest orchids, but even in orchids growing in open habitats with full light conditions [8]. Achlorophyllous orchids are at the extreme point of plant’s nutritional reliance on mycorrhizal fungi, with about 235 species, i.e. more than 50% of all fully mycoheterotrophic plants, that completely lack photosynthetic capability even at maturity, thus being obligately mycoheterotrophic throughout their lifetime [9]. Regarding fungal diversity in orchid mycorrhizae, fully autotrophic orchids generally associate with a wide range of basidiomycetous fungi of the form-genus *Rhizoctonia*, including soil saprotrophs, plant endophytes and pathogens, as well as fungi with poorly known trophic roles [4, 10]. Mixotrophic and fully mycoheterotrophic orchids recruit instead ascomycetes and basidiomycetes having access to large and persistent carbon sources, such as ectomycorrhizal fungal species that simultaneously colonize surrounding tree roots [7, 11]. The latter case constitutes a peculiar tripartite mycorrhizal association in which a shared fungal mycelium transfers photosynthates from the tree to the orchid, thus the orchid indirectly exploiting the tree as a carbon source [12]. Mycorrhizal specificity, represented by the phylogenetic diversity of fungi associated with a particular plant, is low in the majority of green fully photosynthetic orchids, where an orchid species establishes mycorrhizae with several phylogenetically distant fungal species in the *Rhizoctonia* complex [13]. Sometimes protocorms are associated with a smaller range of fungal symbionts than adult plants [5]. Mixotrophic orchids establish either specific or non-specific mycorrhizal associations, sometimes with the coexistence of rhizoctonias and ectomycorrhizal fungi in the same plant species [14–16]. By contrast, the level of specificity is normally very high in achlorophyllous orchids. These orchids often associate with a single fungal clade constituted by a genus or by a lower rank *taxon*, and the fungi involved, with a few exceptions, are not *Rhizoctonia* species [17, 18]. In particular, achlorophyllous orchids from temperate areas of northern latidudes have been shown in several works to associate with non-*Rhizoctonia* ectomycorrhizal fungi that allow the orchid to establish an indirect below ground connection with nearby autotrophic plants. For instance, the Eurasian orchid species *Epipogium aphyllum* has been found associated with basidiomycetes mainly belonging to the ectomycorrhizal genus *Inocybe* [18]. In tropical latitudes instead, achlorophyllous orchids have been recently shown to establish mycorrhizal relationships with non-*Rhizoctonia* saprotrophic fungi. Basidiomycetes belonging to the saprobic Coprinaceae family were found in association with the Asian achlorophyllous orchid *Eulophia zollingeri* [19], *Gymnopus*-related fungal taxa were molecularly identified in the Australian orchid *Erythrorchis cassythoides* [20], litter-decaying *Mycena* in the Caribbean *Wullschlaegelia aphylla* [21] and the Japanese *Gastrodia confusa* [17], while *Marasmius* mycobionts were detected in *Gastrodia sesamoides* from Australia [22]. This peculiar preference for fungal saprotrophs is particularly interesting in tropical orchid species phylogenetically close to temperate orchids, which are instead associated with ectomycorrhizal fungi. For example, the tropical orchid *E. roseum*, showing a very close phylogenetic relationship with the above-mentioned Eurasian species *E. aphyllum*, is associated with saprotrophic Psathyrellaceae [23, 24]. The latter orchid-fungus relationship provides the evidence that the establishment of associations between achlorophyllous orchids and saprobic versus mycorrhizal fungi is not phylogenetically constrained. Environmental factors, such as the warm and humid climate occurring in tropical areas, may allow saprotrophic fungi to extend their growing period and improve their decaying activity on the available organic substrates, thus providing a surplus of nutrients that can be transferred to the fully mycoheterotrophic orchid partners [25]. However, a few exceptions are known. For instance, Roy et al. [26] investigated three Asiatic mycoheterotrophic Neottieae species in Thailand and found that all were associated with ectomycorrhizal fungi, such as Thelephoraceae, Russulaceae and Sebacinales. In a study conducted on fully mycoheterotrophic orchids from sub-tropical Asia, six out of seven analysed orchid species established mycorrhizal relationships with either wood- or litter-decaying saprotrophic fungi, while only one species was associated with ectomycorrhizal fungi [27]. Another exception is represented by the Asian non-photosynthetic orchid species *Chamaegastrodia shikokiana*, which was found to associate with ectomycorrhizal fungi belonging to Ceratobasidiaceae. Mycobionts isolated from *C. shikokiana* were able to form ectomycorrhiza in vitro on seedlings of *Abies firma*, suggesting that the studied orchid may depend on nutrients supplied from the tree species through the hyphae of the mycorrhizal fungi in nature [28].

The present study focused on *C. inverta* (W.W. Smith) Seidenfaden, one of the five achlorophyllous orchid species in the genus *Chameagastrodia*, which is endemic to Yunnan and Sichuan provinces, southern China, usually growing in damp places in montane forests (1200–2600 m a.s.l.) [29]. The aim of this work was to identify the main mycorrhizal partners of *C. inverta* in different plant life stages, by means of morphological and molecular methods. A better understanding of mycorrhizal strategies in orchid species belonging to the genus *Chamaegastrodia*, such as *C. inverta*, would clarify the biology of achlorophyllous orchids and would be of relevance to future in situ or ex situ conservation activities of this endangered species.

## Results

### Mycorrhizal root morphology of *C. inverta*

**Fig. 1:**
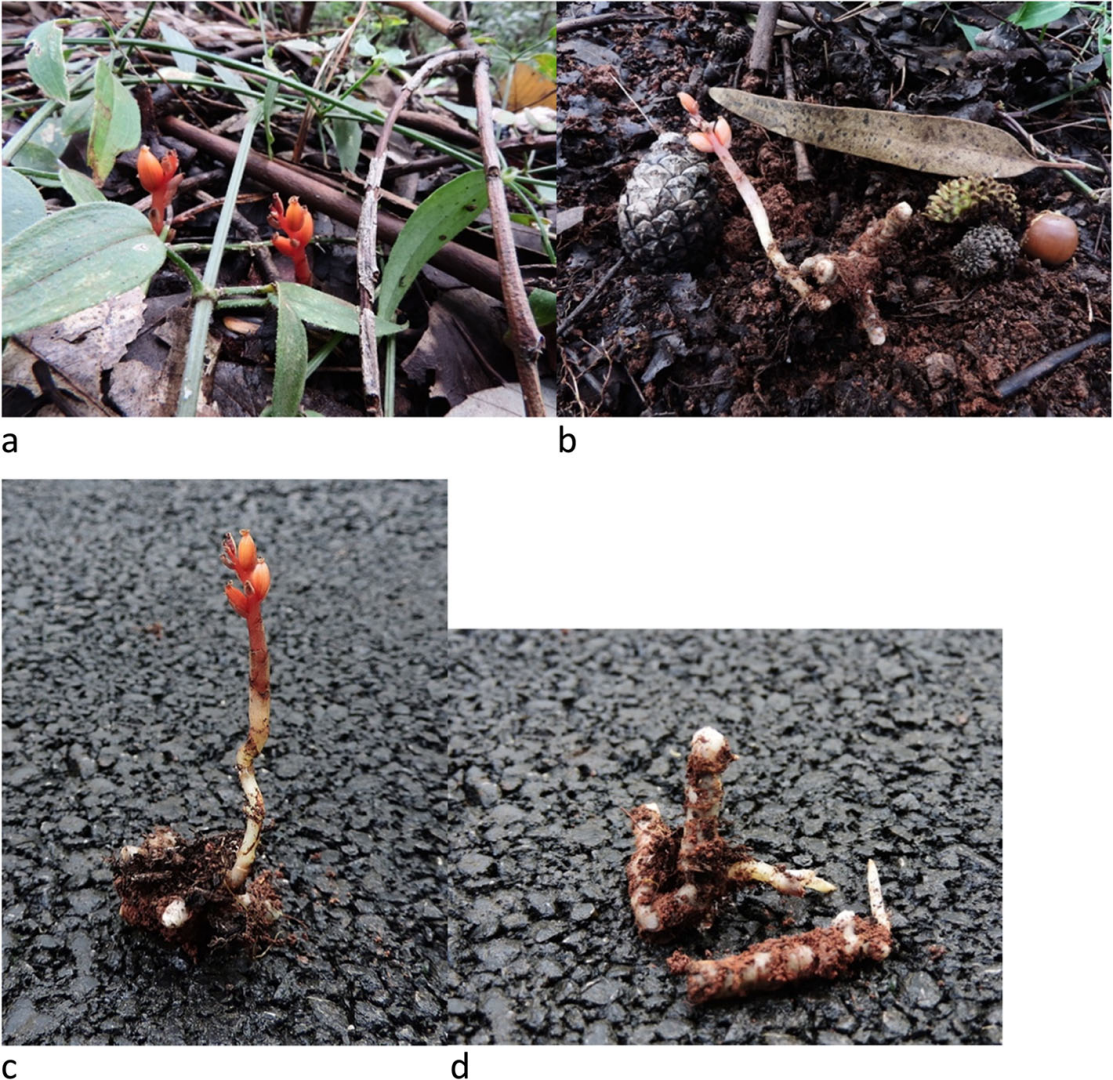
*Chamaegastrodia inverta* adult and completely developed plants (**a**, **b**, **c**) and young hypogeous individuals (seedlings, **d**). Green leaves and stems represented in Fig. 1 **a** belong to non-orchid surrounding vegetation in *C. inverta* habitat

**Fig. 2:**
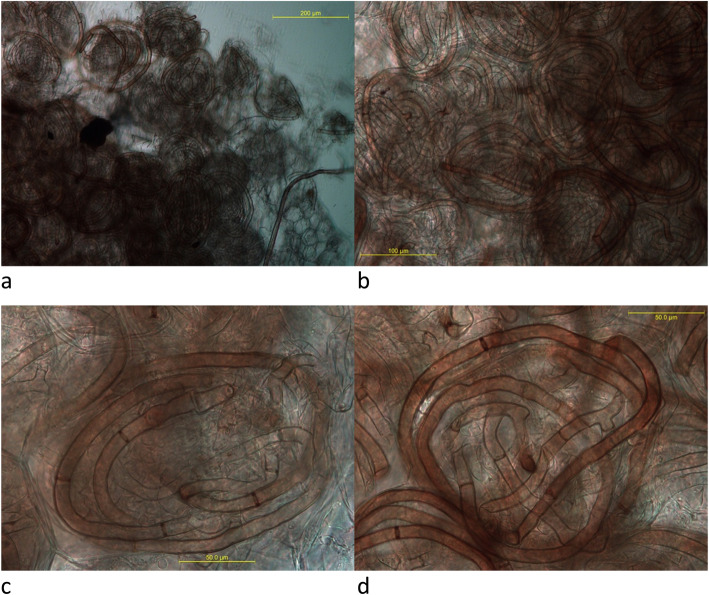
Microscopic characteristics of mycorrhizal roots in *Chamaegastrodia inverta*. **a** Hyphal coils (pelotons) extracted from cortical cells. **b** Fungal pelotons inside orchid root cells. **c**, **d** Details of mycelial morphology characterizing pelotons, with dark, septate, thick-walled hyphae, showing 90° branching, constriction at branch points, and septa near branch origins

Light microscopy on thin sections showed that the roots of all analysed *C. inverta* samples (Fig. 1) were extensively colonized by fungal hyphae. Characteristic dense intracellular hyphal coils (pelotons) were observed in most orchid root cortical cells (Fig. 2a-b). The majority of pelotons appeared intact and completely undigested (Fig. 2a-b). A dominant mycelial morphology characterized the observed pelotons, mainly constituted by dark, septate, thick-walled (7.5–10.5 μm in diameter) hyphae, frequently showing branches produced at right angles to the main hypha, the branch hypha being slightly constricted at the branch origin, and septum often occurring near the branch origin (Fig. 2c-d).

### Fungal isolation

Attempts to isolate in vitro single pelotons extracted from *C. inverta* root cells were in most cases unsuccessful. No hyphal growth was observed from the fungal coils transferred on PDA medium, with a single exception from orchid sample 5. On the contrary, the majority of surface-sterilized root portions yielded fungal mycelia that could be assigned to 32 main morphological types of ascomycetous and zygomycetous fungi by macro- and microscopic observations. These orchid root endophytes were subsequently identified using molecular taxonomy, because the paucity of distinctive morphological characters did not allow a clear taxonomic delimitation, based on microscopy. However, the morphology of isolated mycelia was in most cases completely different from the above-mentioned dominant hyphal morphology observed in the root sections by the microscope.

### Identification of *C. inverta* mycorrhizal symbionts and other endophytes

Molecular analysis allowed the identification of fungi associated with *C. inverta* roots. Sequences were produced from amplicons of both fungal cultures and total root DNA, using the fungal-specific primer pair ITS1-OF/ITS4-OF. These primers successfully yielded sequences from all the 32 fungal isolates and the 8 analysed plants. Amplicon electrophoretic profiles displayed high intensity bands from 600 to 800 bp. The general primer pair ITS1F/ITS4 gave, instead, less consistent amplification, and in some cases, never produced any amplicon, or just amplified the orchid plant DNA (data not shown).

Total root DNA sequencing revealed that mycorrhizal tissue was dominated by fungi belonging to the family Ceratobasidiaceae (Table 1). For 7 out of the total 8 investigated *C. inverta* plants, amplified sequences were related to *Ceratobasidium* sequences in GenBank. The young orchid plant (seedling) sample 6 yielded sequences 6a (accession no. MT278316) and 6b (MT278317) with close identity to GenBank accession sequences of Russulaceae and Ceratobasidiaceae, respectively (Table 1).

**Table 1:** Fungal diversity molecularly detected in *Chamaegastrodia inverta* roots, from extracted total root DNA

***C. inverta*** sample	GenBank code	Best BLAST match(es)	Accession code	Overlap length	% match
1b (adult)	MT278309	Vouchered mycorrhizae (Basidiomycota)	AB303058	1160	98%
		Uncultured *Ceratobasidium*	JQ991678	1096	99%
2b (adult)	MT278310	Vouchered mycorrhizae (Basidiomycota)	AB303057	1162	98%
		Uncultured *Ceratobasidium*	JQ991678	1096	99%
		*Ceratobasidium* sp.	GQ175299	1081	96%
3b (adult)	MT278311	Vouchered mycorrhizae (Basidiomycota)	AB303057	1188	98%
		*Ceratobasidium* sp.	GQ175299	1122	96%
4a (adult)	MT278312	Vouchered mycorrhizae (Basidiomycota)	AB303058	1155	98%
		Uncultured *Ceratobasidium*	JQ991678	1090	99%
		*Ceratobasidium* sp.	GQ175299	1068	95%
4b (adult)	MT278313	Vouchered mycorrhizae (Basidiomycota)	AB303058	2055	98%
		Uncultured *Ceratobasidium*	JQ991678	1994	99%
		*Ceratobasidium* sp.	GQ175299	1920	96%
5a (adult)	MT278314	Vouchered mycorrhizae (Basidiomycota)	AB303058	1088	96%
		Uncultured *Ceratobasidium*	JQ991678	1029	97%
		*Ceratobasidium* sp.	GQ175299	1002	94%
5b (adult)	MT278315	Vouchered mycorrhizae (Basidiomycota)	AB303057	1129	97%
		Uncultured *Ceratobasidium*	JQ991678	1062	98%
		*Ceratobasidium* sp.	GQ175299	1048	95%
6a (seedling)	MT278316	*Russula cerolens*	KX095042	1203	98%
6b (seedling)	MT278317	Vouchered mycorrhizae (Basidiomycota)	AB303058	1138	98%
		Uncultured *Ceratobasidium*	JQ991678	1079	98%
7a (seedling)	MT278318	Vouchered mycorrhizae (Basidiomycota)	AB303058	1155	98%
		Uncultured *Ceratobasidium*	JQ991678	1090	99%
		*Ceratobasidium* sp.	GQ175299	1068	96%
8a (seedling)	MT278319	*Phomopsis* sp.	KF428571	1161	99%

BLAST search closest matches of fungal internal transcribed spacer DNA sequences amplified from *C. inverta*. Sample GenBank accession codes, accession codes for the closest GenBank matches, sequence identity, and overlap of each match are reported

**Fig. 3:**
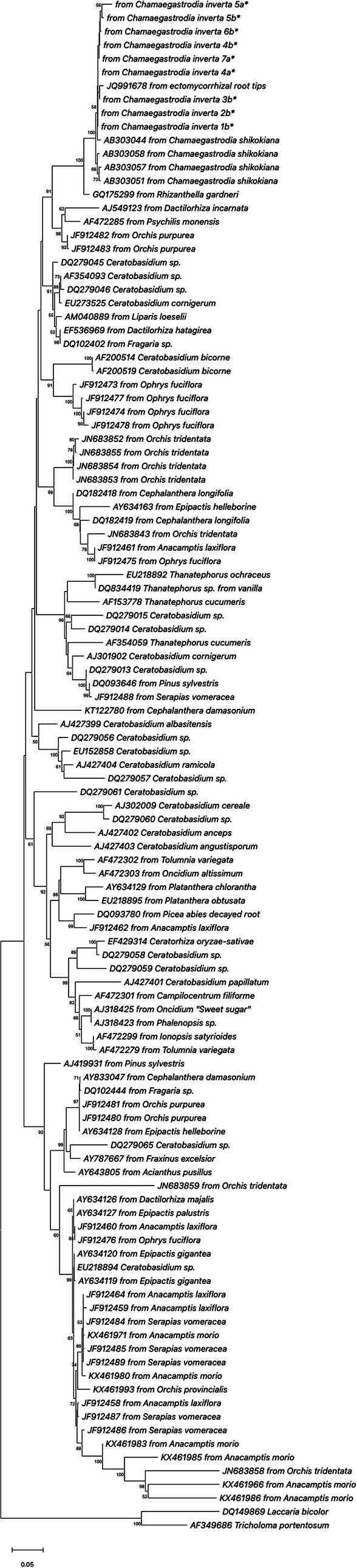
Neighbour-joining phylogenetic tree showing the relationship between the Ceratobasidiaceae sequences obtained from *Chamaegastrodia inverta* (*) and selected database relatives. Kimura 2-parameter distances were used. Bootstrap values are based on percentages of 1000 replicates. The tree was rooted with *Laccaria bicolor* and *Tricholoma portentosum* as outgroups

**Fig. 4:**
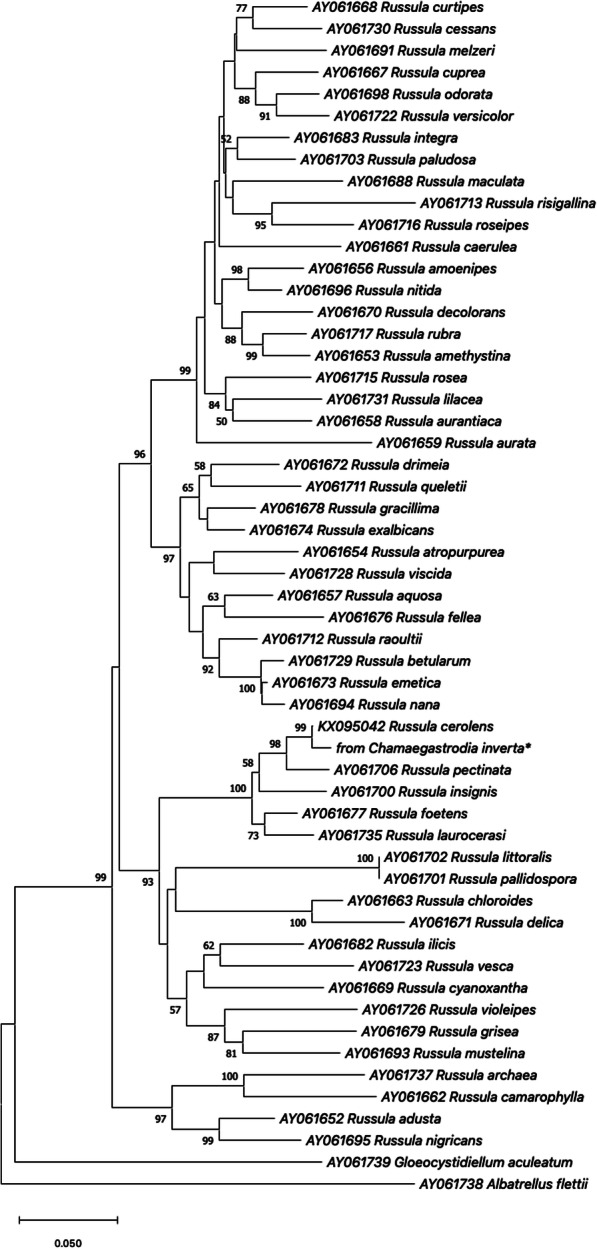
Neighbour-joining phylogenetic tree showing the relationship between the *Russula* sequence obtained from *Chamaegastrodia inverta* (*) and selected database relatives. Kimura 2-parameter distances were used. Bootstrap values are based on percentages of 1000 replicates. The tree was rooted with *Gloeocystidiellum aculeatum* and *Albatrellus flettii* as outgroups

Phylogenetic analysis clarified the relationships of *C. inverta* root-associated fungi within Russulaceae and Ceratobasidiaceae. Sequences retrieved from the studied orchid plants could be aligned with fungal sequences from various orchid and non-orchid plant hosts, isolated strains, and sporophores. A neighbour-joining tree revealed that all *Ceratobasidium*-like sequences obtained in this study clustered into a single well-supported clade (Fig. 3) and are closely related to ceratobasidioid fungi previously found in the rhizome of *Chamaegastrodia shikokiana* in Japan and in ectomycorrhizal root tips in China. These fungi formed a clade with 100% bootstrap support with another member of Ceratobasidiaceae isolated from the Australian mycoheterotrophic orchid *Rhizanthella gardneri* (Fig. 3). The phylogenetic tree from the Russulaceae dataset showed that the sequence amplified from roots of *C. inverta* plant sample 6 fell in a cluster including *Russula cerolens* (that was the closest match) *R. pectinata* and *R. insignis* (Fig. 4).

Apart from these basidiomycetes identified from total root DNA, sequences of ascomycetous and zygomycetous fungi were obtained from the isolated strains (Table 2). Among them, sequences corresponding to the genus *Mortierella*, in the Mortierellaceae family of Mucoromycota were most commonly recovered from 46.8% of cultured mycelia, including the sole strain obtained from peloton (sample 5, accession no. MT278348), whereas 25% of isolated fungi yielded sequences with very high ITS similarity to *Phomopsis* species, belonging to the family Diaporthaceae of Ascomycota (Table 2). In the latter family was also found the best match for sequences obtained from 18.7% of isolates, belonging to the genus *Diaporthe*. Other ascomycetes from the genera *Leptodontidium* (Leptodontidiaceae family) and *Chaetomium* (Chaetomiaceae) were also sporadically found (Table 2).

**Table 2:** Fungal diversity molecularly detected in *Chamaegastrodia inverta* roots, from DNA extracted from isolated fungi

C. inverta sample	GenBank code	Best BLAST match(es)	Accession code	Overlap length	% match
1 (1) a	MT278320	Uncultured fungus	AB520571	1184	99%
		*Mortierella alpina*	KJ469836	1168	98%
1 (1) c	MT278321	Uncultured fungus	AB520571	1194	99%
		*Mortierella alpina*	KJ469836	1177	99%
1 (1) d	MT278322	Uncultured fungus	AB520571	1203	99%
		Uncultured *Mortierella*	HG936573	1182	98%
1 (1) e	MT278323	Uncultured fungus	AB520571	1994	99%
		Uncultured *Mortierella*	HG936573	1965	98%
1 (2) a	MT278324	Uncultured fungus	AB520571	1190	99%
		Uncultured *Mortierella*	HG936573	1170	98%
1 (2) c	MT278325	Uncultured fungus	AB520571	1229	99%
		Uncultured *Mortierella*	HG936573	1208	98%
1 (2) e	MT278326	Uncultured fungus	AB520571	1214	99%
		Uncultured *Mortierella*	HG936573	1199	99%
1 (2) g	MT278327	Fungal sp. strain	KU838618	1033	99%
		*Phomopsis* sp.	KF428571	1033	99%
1 (3)	MT278328	Uncultured fungus	AB520571	1190	98%
		Uncultured *Mortierella*	HG936573	1173	98%
1 (3) a	MT278329	Uncultured fungus	KC222700	1037	98%
		*Mortierella* sp.	KX640303	1029	99%
1 (3) b	MT278330	Uncultured fungus	AB520571	1210	99%
		Uncultured *Mortierella*	HG936573	1192	98%
1 (4)	MT278331	*Phomopsis* sp.	KF428571	1050	99%
1 (6) a	MT278332	*Phomopsis* sp.	KF428571	1040	99%
1 (6) b	MT278333	Uncultured fungus	AB520571	1218	99%
		Uncultured *Mortierella*	HG936573	1199	98%
1 (6) c	MT278334	*Phomopsis* sp.	KF428571	1055	99%
3 (3) b	MT278335	Uncultured fungus	KC222831	977	98%
		Diaporthales sp.	KF428606	972	99%
		*Diaporthe* sp.	FJ799941	952	96%
3 (3) c	MT278336	Uncultured fungus	KC222831	977	98%
		Diaporthales sp.	KF428606	972	99%
		*Diaporthe* sp.	FJ799941	946	96%
3 (3) d	MT278337	Diaporthales sp.	KF428606	972	99%
		*Diaporthe* sp.	FJ799941	944	96%
3 (3) e	MT278338	Diaporthales sp.	KF428606	972	99%
		*Diaporthe* sp.	FJ799941	950	96%
3 (4)	MT278339	Uncultured fungus	KF296769	1127	98%
		Uncultured *Leptodontidium*	JF519497	1127	98%
3 (4) b	MT278340	Uncultured fungus	KF296769	1133	98%
		Uncultured *Protoventuria*	JQ346991	1133	98%
		Uncultured *Leptodontidium*	JF519497	1133	98%
4 (3) b	MT278341	Diaporthales sp.	KF428606	1002	100%
		*Diaporthe* sp.	FJ799941	979	97%
4 (3) c	MT278342	Uncultured fungus	AB520571	1195	99%
		Uncultured *Mortierella*	HG936573	1179	98%
4 (3) d	MT278343	Diaporthales sp.	KF428606	1002	100%
		*Diaporthe* sp.	FJ799941	987	97%
5 (1) a	MT278344	*Phomopsis* sp.	KF428571	1048	99%
5 (1) b	MT278345	*Phomopsis* sp.	KF428571	1042	99%
5 (3)	MT278346	Uncultured fungus	AB520571	1177	98%
		*Mortierella alpina*	KJ469836	1164	98%
5 (4) a	MT278347	*Chaetomium nigricolor*	JF439467	1057	99%
5 (Pel)	MT278348	Uncultured fungus	AB520571	1190	98%
		Uncultured *Mortierella*	HG936573	1173	98%
7 (2) a	MT278349	Fungal endophyte isolate	KR015900	1026	98%
		*Phomopsis* sp.	JQ341094	1020	99%
7 (2) b	MT278350	Fungal endophyte isolate	KR015900	1022	98%
		*Phomopsis* sp.	JQ341094	1020	98%
8 (1)	MT278351	Uncultured fungus	AB520586	479	84%
		Uncultured *Mortierella*	FJ197919	473	83%

BLAST search closest matches of fungal internal transcribed spacer DNA sequences amplified from *C. inverta*. In sample codes, first numbers (from 1 to 8) indicate the plant samples, numbers in brackets the root portions, letters the isolated fungal strain. Sample GenBank accession codes, accession codes for the closest GenBank matches, sequence identity, and overlap of each match are reported

## Discussion

This study represents the first assessment of root fungal diversity in the rare, cryptic, and narrowly distributed Chinese orchid species *Chamaegastrodia inverta*. Microscopic analysis, showing the heavy presence of fungal pelotons in the root cortical cells of all studied orchid samples, provided the first evidence of the establishment of mycorrhizal associations in *C. inverta*. The observed extent of fungal colonization in the orchid mycorrhizal roots was not surprising, given the achlorophyllous condition of the investigated plant species. The studied orchid was expected to be wholly mycoheterotrophic and therefore highly dependent on soil fungi for its nutrition, based on results showed from the previously studied member of the genus *Chamaegastrodia*, *C. shikokiana* in Japan [28], and the general mycorrhizal condition of non-photosynthetic forest orchids [1, 30].

The use of different experimental approaches, including morphological analysis combined with molecular sequencing, following both culture-dependent methods and direct total orchid root DNA amplification, allowed the detection and identification of *C. inverta* root-associated fungi, in different plant life stages. Results showed that the primary mycorrhizal symbionts of *C. inverta* are within the family Ceratobasidiaceae. This family is included in the diverse group of *Rhizoctonia*-like fungi, which comprises a range of rather distantly related fungal *taxa* characterised by homogenous asexual stage hyphal morphology (90° branching of hyphae, a constriction at the branch point, and a septum near the point of origin in the branch hyphae), as well as by a common significant predisposition to establish mycorrhizal symbiosis with orchids [4, 31, 32]. The morphology of mycelia forming pelotons in the root cells of analysed *C. inverta* individuals, showing typical *Rhizoctonia* features, is consistent with molecular identification of Ceratobasidiaceae fungal symbionts. Ceratobasidioid fungi have been previously found to associate with several other orchids including both tropical and temperate species [32–34]. Members of this fungal family have been recognized as important associates in epiphytic orchids belonging to different genera, such as *Oncidium* [35], *Ionopsis* and *Tolumnia* [36, 37], as well as in terrestrial orchids, of both forest and meadow habitats, including *Goodyera* [38–40], *Anacamptis*, *Cephalanthera* and *Orchis* [16, 34, 41]. Although mycorrhizal associations with Ceratobasidiaceae involve orchids with very different biogeographical and ecological features, the great majority of orchid species that have been found to establish a trophic relationship with Ceratobasidiaceae fungi belong to the same physiological category of green orchids, including species with different degrees of photosynthetic capability, from fully autotrophic to mixotrophic species [8, 42, 43]. Achlorophyllous non-photosynthetic orchids, instead, are almost completely excluded from mycorrhizal partnerships with Ceratobasidiaceae, with very few exceptions [28, 44]. Our finding of ceratobasidioid fungi as dominant associates in the roots of the achlorophyllous forest orchid *C. inverta* represents a new record of the rare association between the identified fungal group and fully mycoheterotrophic orchids in nature. Phylogenetic relationships reconstructed from rDNA sequence information suggest that the *C. inverta* associated Ceratobasidiaceae have limited genetic diversity and likely belong to the same species (Fig. 3). *Chamaegastrodia inverta* mycobionts are phylogenetically close to a peculiar group of Ceratobasidiaceae showing ectomycorrhizal capability, such as the fungi previously found in *C. shikokiana* in Japan, which were also able to form ectomycorrhizas on the rootlets of the woody plant species *Abies firma* sedlings in pot culture [28], and the fungi associated with the Australian subterranean orchid (flowering below ground) *Rhizanthella gardneri* [44]. Mycorrhizal fungi, isolated from pelotons extracted from the rhizomes of the latter orchid species, were tested by Bougoure and collaborators for their ability to form ectomycorrhizal associations with several plant species belonging to the genus *Melaleuca*, which resulted in the undoubted formation of mantle and Hartig net, typical ectomycorrhizal structures [44]. Ectomycorrhizal taxa constitute a rare exception among Ceratobasidiaceae, the great majority of members within this fungal family, as well as *Rhizoctonia*-forming Agaricomycotina in general, being regarded as saprotrophs and plant pathogens [28, 31, 43–45]. The previously reported cases of tripartite relatioships *C. shikokiana*-*Ceratobasidium*-*A. firma* [28] and *R. gardneri*-*Ceratobasidium*-*Melaleuca* [44] involved ceratobasidioid fungi which were able to establish orchid mycorrhizas with fully mycoheterotrophic orchid species and ectomycorrhizas with autotrophic tree or shrub hosts simultaneously, the photosynthetic plant partner being the provider of carbon for the system. An additional example of ectomycorrhizal Ceratobasidiaceae was provided by the fungi isolated from the roots of the mixotrophic orchid *Platanthera minor*, which were also found to show ectomycorrhiza-forming ability on *Pinus densiflora*, the sole ectomycorrhizal tree species in the sampling sites in Japan [45]. The *P. minor* associated *Ceratobasidium* fungi formed a highly supported clade with mycobionts from *C. shikokiana* and *R. gardneri* in the phylogenetic analyses performed by Yagame et al. [45]. The Ceratobasidiaceae associated with *C. inverta*, in this study, are also closely related and cluster with the above mentioned ectomycorrhizal ceratobasidioid fungi from *C. shikokiana* and *R. gardneri*. It is therefore possible that *C. inverta* establishes a tripartite relationship with some of the surrounding autotrophic plants in the forest habitats where it grows, using the ceratobasidioid associated fungi to exploit the ectomycorrhizal plant as a carbon source. Further studies are necessary to test this hypothesis, by determining *C. inverta* natural abundance in ^13^C and ^15^N compared with those of surrounding photosynthetic plants, in order to confirm and quantify the contribution of fungal source to the orchid carbon nutrition [46]. Besides, experiments of ectomycorrhiza formation on roots of potential tree hosts available in *C. inverta* habitats, using Ceratobasidiaceae isolated from the studied orchid as inoculum, would clarify the ability of the orchid fungal associates to establish ectomycorrhizal symbiosis with surrounding autotrophic partners. Our attempt of fungal isolation from pelotons extracted from the cortical cells of *C. inverta* roots was unsuccessful. The sole strain observed to apparently grow from a peloton isolated from orchid sample 5 was molecularly similar to an uncultured fungus detected in agricultural soil from Japan [47] and to a *Mortierella* sequence amplified from *Zea mays* field soil samples in Germany [48] (Table 2). This isolated mycelium may actually reflect a fungal contaminant from spore or a non-mycorrhizal root endophyte from hyphal fragment accidentally trapped in the cultured peloton. The difficulty in isolating in axenic culture the real *C. inverta* fungal symbionts from pelotons was confirmed by the result of isolation attempts from root fragments, which provided a number of ascomycetous and zygomycetous strains, but did not yield any ceratobasidioid basidiomycete. This result is in agreement with previous studies on *C. shikokiana* and *R. gardneri*, involving similar Ceratobasidiaceae mycorrhizal fungi. In the former study on *C. shikokiana* the authors reported that no fungal growth was observed on Czapek-Dox medium, which they normally used for saprobic fungi isolation, while active mycelial growth was obtained from pelotons cultured on a Modified Melin-Norkrans medium, specific for ectomycorrhizal fungi [28]. Similarly, in the study on *R. gardneri*, Bougoure et al. [44] found that isolation and growth of fungal pelotons from the roots of the studied orchid were mostly unsuccessful, with extracted hyphal coils failing to grow or colonies suddenly dying after initial growth. The absence of hyphal growth from *C. inverta* mycobiont extracted pelotons on PDA medium suggests that the analysed fungi belong to the ectomycorrhizal trophic group and may require very specific media to be isolated. However, the ecology and lifestyle of Ceratobasidiaceae associated with achlorophyllous mycoheterotrophic orchids is difficult to predict and generalize, and requires in-depth analyses to be understood, in every single association with different plant species. For instance, Bougoure et al. [49] showed that Ceratobasidiaceae associated with *R. gardneri* obtained carbon by both saprothrophic and mycorrhizal means, simultaneously. In the latter work, isotopically labelled tracers, ^13^CO_2_ and double-labelled [^13^C-^15^N]glycine were used to assess the direction of carbon and nitrogen transfers between the plants involved in the investigated tripartite association via the fungal connections, showing that *R. gardneri* obtained nutrients from the associated mycorrhizal ceratobasidioid fungi, which were able to derive carbon not only from surrounding autotrophic shrubs via ectomycorrhizas, but also from soil organic matter via saprotrophic activity [49].

One out of the three analysed *C. inverta* young seedlings, sample 6, showed an interesting association with another basidiomycete (Fig. 4) highly similar to *Russula cerolens* in GenBank from an unpublished study by Ma et al. on Russulaceae diversity in Heilongjiang Province, in China, and to sequences from specimens of *R. insignis* and *R. pectinata* collected in Europe [50]. The amplified *Russula* mycorrhizal fungus was co-occurring with *Ceratobasiaceae* in the roots of the same *C. inverta* individual (Table 1). Species in the large mushroom genus *Russula* are well documented as ecologically important ectomycorrhizal symbionts with forest tree species [50, 51]. *Russulaceae* fungi have been found to be mycorrhizal partners of a variety of orchids belonging to different physiological types, such as the fully mycoheterotrophic species *Corallorhiza maculata* [11] and *Dipodium hamiltonianum* [52], the partially mycoheterotrophic orchids *Epipactis microphylla* [7] and *Limodorum abortivum* [53], as well as green photosynthetic *Cypripedium* species [54]. In a comprehensive study on mycorrhizal diversity in the fully mycoheterotrophic orchid genus *Hexalectris*, Kennedy et al. [55] found that *H. brevicaulis* collected in Mexico and *H. grandiflora* from USA displayed mixed associations with Russulaceae and Sebacinaceae symbionts. To our knowledge the simultaneous presence of ectomycorrhizal *Russulaceae* and *Ceratobasidiaceae* in the same achlorophyllous orchid species represents a new finding from the present work, where the two mycorrhizal fungi were even detected in the same *C. inverta* individual. This result supports the hypothesis of *C. inverta* preference for ectomycorrhizal fungi to establish trophic relationships that may also involve other plants interconnected by underground fungal network. This specificity toward ectomycorrhizal fungal partners needs to be confirmed with additional analyses on *C. inverta* samples from different localities. It is interesting that the presence of *Russula* in *C. inverta* roots was only detected at young seedling stage, while all analysed adult plants only yielded Ceratobasidiaceae sequences. It is possible that the analysed orchid species associates with different fungi during different stages of its development, as it was previously shown in a variety of orchids characterized by changing level of mycorrhizal specificity through life stages, from germinating seeds and protocorms to adult plants [5, 30]. Experiments using orchid seed baits buried in sites characterized by the presence of *C. inverta* populations may be crucial to identify fungal taxa able to stimulate seed germination and to sustain protocorm development in nature [56], as well as to clarify whether or not the identity of mycorrhizal fungi associating with *C. inverta* can be affected by the plant life stage.

Besides typical orchid mycorrhizal taxa, some other fungi with unclear functional roles were also detected in the studied orchid roots. They were mainly uncovered using the culture-dependent approach, thus demonstrating the importance of culture-based method for the accurate analysis of fungal community associated with orchid roots (Table 2). Among them, the most abundant were in the Mucoromycota, which represent an exceptional report as orchid associates, and were previously considered root pathogens or saprotrophic fungi in orchid dead cells [57]. Ascomycota from different families were more sporadically detected, the best matches for sequences amplified from *C. inverta* being fungi from various sources, including *Phomopsis* sp. from roots of *Populus* trees (Bonito et al. unpublished), *Diaporthe* sp. from leaves of *Ipomoea philomega* [58], uncultured *Leptodontidium* from roots of *Fagus sylvatica* (Schnecker et al. unpublished), and *Chaetomium nigricolor* from forest soil in Zijin Mountain, China [59] (Table 2). These ascomycetes may represent root fungal endophytes [43, 60]. However, some of them, such as *Leptodontidium* fungi, are very frequently selected during in vitro isolation or PCR amplification from orchid tissues using fungus-specific primers [34, 61, 62]. Further physiological characterization of these fungi is needed to understand whether they play any trophic role in their association with *C. inverta*.

## Conclusions

Our results provide new insights on the spectrum of orchid-fungus symbiosis suggesting an unprecedented mixed association between the achlorophyllous forest orchid *C. inverta* and ectomycorrhizal fungi belonging to Ceratobasidiaceae and Russulaceae. This urges for more in-depth investigations of mycorrhizal diversity and physiology in the five orchid species belonging to the genus *Chamaegastrodia*, which may represent useful models to understand the evolution and specificity of mycoheterotrophic interactions, even if their rarity constitutes a challenge for performing detailed, large scale experiments.

## Methods

### Orchid collection

*Chameagastrodia inverta* root samples were collected in September 2015, from a forest with coniferous species of *Pinus*, mixed with various broad-leaved trees dominated by *Eucalyptus* sp. and *Quercus* sp., located in the western part of Kunming Botanical Garden, Kunming Institute of Botany, Chinese Academy of Sciences, Kunming, Yunnan Province, China. No permissions were necessary to collect plant samples, using a protocol that allows plant survival. Plants were identified by the authors. After harvesting the root material necessary for the study, plants were replaced in the sampling site, in the exact location where they were found. In total, eight orchid individuals were sampled, including 5 adult and completely developed plants, at the end of flowering stage, and 3 hypogeous young plants (seedlings) at an initial stage of development (Fig. 1a-d). Although the sampling size for the studied species was limited due to its rarity, small population size and the cryptic nature of its inflorescence, which is the only above ground organ, the investigated site provided a significant number of samples representing different life stages of the analysed orchid. All collected roots were washed under running water and carefully brushed in order to remove soil debris. Mycorrhizal morphology of fresh root samples was observed on thin cross-sections under a light microscope at 100 to 1000-fold magnification. Root fragments exhibiting high fungal colonization were immediately processed for fungal isolation. Part of root material was frozen in liquid nitrogen and stored at − 80 °C for molecular analysis.

### Fungal isolation

Isolation of fungi from fresh orchid root fragments was performed immediately after sampling. Single pelotons (hyphal coils within orchid root cells) were dissected from the outer cortex of *C. inverta* roots following Rasmussen [4], rinsed twice in double distilled water, transferred in 1 μl water, and cultured on potato dextrose agar (PDA, Solarbio, Beijing, China) for both morphological and molecular identification.

Fungi were also isolated from root segments [41] surface-sterilized with consecutive washes of 1:5 sodium hypochlorite (30 s), rinsing in three changes of sterile water, cut in 3–5 mm long pieces, and cultured on PDA. Petri dishes were incubated at room temperature (20–25 °C) in the dark for up to 2 months to allow the development of slow-growing mycelia.

### Molecular identification of root fungal associates

Both DNA from isolated fungi and total DNA from orchid root samples were extracted following the cetyltrimethyl ammonium bromide (CTAB) method [63].

Fungal ITS regions were amplified by polymerase chain reaction (PCR), using the primer pairs ITS1F/ITS4 [64] and ITS1-OF/ITS4-OF [65] in 50 μL reaction volume, containing 38 μL steril distilled water, 5 μL 10 × buffer (100 mM Tris-HCl pH 8.3, 500 mM KCl, 11 mM MgCl2, 0.1% gelatin), 1 μL of dNTP mixture of 10 mM concentration, 0,25 μM of each primer, 1.5 U of RED TaqTM DNA polymerase (Sigma) and approximately 10 μg of extracted genomic DNA. Amplifications were performed in a PerkinElmer/Cetus DNA thermal cycler, under the following thermal conditions: 1 cycle of 95 °C for 5 min initial denaturation before thermocycling, 30 cycles of 94 °C for 40 s denaturation, 45 s annealing at various temperatures following Taylor & McCormick [65], 72 °C for 40 s elongation, followed by 1 cycle of 72 °C for 7 min extension. The resulting PCR products were electrophoresed in 1% agarose gel with ethidium bromide and purified with the QIAEX II Gel Extraction Kit (QIAGEN) following the manufacturer’s instructions. Controls with no DNA were included in every amplification experiment in order to test for the presence of laboratory contamination from reagents and reaction buffers.

DNA sequencing was performed at the GENEWIZ Company, Tianjin, China.

Sequences were edited, assembled using the program Sequencher 4.1 for MacOS 9, and analysed with BLAST searches against the National Center for Biotechnology Information (NCBI) sequence database (GenBank) [66]. Fungal DNA sequences amplified from *C. inverta* were submitted to GenBank under accessions MT278309 - MT278351.

Phylogenetic analysis was conducted with Mega v. 5.0 [67]. DNA sequences were aligned with Clustal X v. 2.0 [68] and neighbour-joining trees against selected database sequences were constructed using Kimura 2-parameter distances, with bootstrapping of 1000 replicates [69]. Distinct phylogenetic analyses were performed for the phylogenetically distant fungi identified from the roots of investigated orchids. The ceratobasidioid fungi tree was rooted with *Laccaria bicolor* and *Tricholoma portentosum*, while *Gloeocystidiellum aculeatum* and *Albatrellus flettii* were used as outgroups to root the *Russula* fungi tree.

## Data Availability

The Fungal DNA sequences amplified during this study are available in GenBank under accessions MT278309 - MT278351. No permissions were necessary to collect plant samples, using a protocol that allows plant survival. Plants were identified by the authors. After harvesting the root material necessary for the study, plants were replaced in the sampling site, in the exact location where they were found.

## Abbreviations

CTAB: Cetyltrimethyl ammonium bromide
PCR: Polymerase chain reaction
